# Coordinated Dual-Fin Actuation of Bionic Ocean Sunfish Robot for Multi-Modal Locomotion

**DOI:** 10.3390/biomimetics10080489

**Published:** 2025-07-24

**Authors:** Lidong Huang, Zhong Huang, Quanchao Liu, Zhihao Song, Yayi Shen, Mengxing Huang

**Affiliations:** 1 School of Information and Communication Engineering, Hainan University, Haikou 570228, China; huangld2021@hainanu.edu.cn (L.H.); liuqc06@163.com (Q.L.); szh794@hainanu.edu.cn (Z.S.); 2 State Key Laboratory of Marine Resources Utilization in South China Sea, Hainan University, Haikou 570228, China; 3 College of Mechanical & Electrical Engineering, Nanjing University of Aeronautics and Astronautics, Nanjing 210016, China; yayi.shen@nuaa.edu.cn

**Keywords:** robotic fish, vertical dual-fin, coordinated actuation, multi-modal locomotion, 3-D maneuverability

## Abstract

This paper presents a bionic dual-fin underwater robot, inspired by the ocean sunfish, that achieves multiple swimming motions using only two vertically arranged fins. This work demonstrates that a mechanically simple platform can execute complex 2-D and 3-D motions through advanced control strategies, eliminating the need for auxiliary actuators. We control the two fins independently so that they can perform cooperative actions in the water, enabling the robot to perform various motions, including high-speed cruising, agile turning, controlled descents, proactive ascents, and continuous spiraling. The swimming performance of the dual-fin robot in executing multi-modal locomotion is experimentally analyzed through visual measurement methods and onboard sensors. Experimental results demonstrate that a minimalist dual-fin propulsion system of the designed ocean sunfish robot can provide speed (maximum cruising speed of 1.16 BL/s), stability (yaw amplitude less than 4.2°), and full three-dimensional maneuverability (minimum turning radius of 0.89 BL). This design, characterized by its simple structure, multiple motion capabilities, and excellent motion performance, offers a promising pathway for developing robust and versatile robots for diverse underwater applications.

## 1. Introduction

Underwater robots play a crucial role in marine development and underwater activities, offering broad application prospects and great potential value. The bio-inspired underwater robots, in particular, offering significant advantages in efficiency, agility, and minimal fluid disturbance over traditional underwater vehicles [1,2]. Research has primarily focused on two dominant propulsion modes found in nature: body and/or caudal fin (BCF) locomotion, which uses body undulation and/or a tail fin [3,4], and medial and/or pair fin (MPF) locomotion, which relies on pectoral and/or other paired fins [5,6].

A primary limitation of many robotic fish is their insufficient cruising speed and inherent instability. Increasing speed often necessitates more powerful actuators or novel propulsion mechanisms, such as the high-speed bionic dolphin that can even achieve water-leaping [7,8], or the use of multi-joint flexible bodies to improve propulsive efficiency [9,10,11]. Concurrently, the oscillatory nature of flapping-based propulsion can induce significant yaw instability, compromising the robot’s ability to serve as a stable platform for sensors like cameras. To mitigate this, some designs have explored unique multi-fin configurations, such as the dual-caudal-fin robot by Zhang et al. [12,13], which improved stability but still wrestled with the fundamental trade-offs between speed, stability, and system complexity.

Achieving agile maneuverability, especially in the vertical plane, presents another significant hurdle. Many platforms achieve 3-D motion by adding dedicated auxiliary control mechanisms, which fundamentally increases mechanical complexity. Examples include incorporating pectoral fins for pitch and roll control [14], using multi-fin systems where each fin’s attack angle is independently controlled [15], relying on retractable pistons as seen in the So-Fi robot for coral reef exploration [16], or other actuators [17]. Recent explorations into hybrid-driven systems [18,19] and gliding-capable robots with underactuated tails [20] further illustrate the field’s effort to enhance maneuverability, yet often at the cost of a more intricate and potentially less reliable mechanical structure.

Parallel to hardware development, researchers have employed a variety of advanced control strategies to enhance performance. Central Pattern Generators (CPGs) are widely used to generate rhythmic swimming gaits [5,9]. Deep Reinforcement Learning (DRL) has emerged as a powerful tool for optimizing swimming efficiency and path-following in complex environments [4,21,22,23]. Furthermore, model-based predictive control, leveraging data-driven dynamic models, has shown success in improving the speed and stability of flexible-tailed robots [24,25]. However, a crucial observation is that these sophisticated control algorithms are often applied to mechanically complex systems, essentially using software complexity to manage hardware complexity, rather than addressing the root issue at the level of core mechanical design.

This review of existing challenges highlights the need for a new design paradigm that can simultaneously address speed, stability, and maneuverability without resorting to mechanical or control complexity. To this end, we turn to a unique biological exemplar: the ocean sunfish (*Mola mola*). Unlike conventional swimmers, the ocean sunfish generates thrust through the oscillation of its large, vertically-opposed dorsal and anal fins [26]. This vertical dual-fin configuration offers a profound biomechanical advantage: it provides an intrinsic capacity for generating direct vertical thrust and yaw moments using the same actuators responsible for forward propulsion. This suggests a pathway to a unified solution: a structure inherently stable due to its symmetrical flapping, powerful due to the large fin surfaces, and highly maneuverable due to the vertical force vectoring capability.

Inspired by this biological principle, this paper presents the design, fabrication, and validation of a bionic ocean sunfish robot that embodies a minimalist design philosophy. Our robot utilizes only two actuators and no auxiliary control mechanisms. We demonstrate that by implementing specific coordinated dual-fin actuation strategies, this mechanically simple platform can comprehensively address the aforementioned challenges. It achieves a high cruising speed (1.16 BL/s), maintains exceptional postural stability (yaw amplitude < 4.2°), and exhibits a full suite of 2-D and 3-D multi-modal locomotion, including agile turning (minimum turning radius of 0.89 BL), controlled descents, and proactive ascents from a negatively buoyant state. The work herein validates that a properly engineered bio-inspired design can overcome the conventional trade-offs, offering a new pathway for robust, high-performance, and versatile underwater robots.

The main contributions of this study can be summarized as follows:

1. We present the design and validation of an ocean sunfish-inspired robotic fish that is propelled by only two actuators and requires no auxiliary mechanisms. This contribution demonstrates the feasibility of achieving function-structure decoupling by imitating a specific biological architecture.
2. We systematically showcase the multi-modal locomotion performance of the robot, particularly its high stability, agility, 3-D maneuverability, and its signature capability for proactive ascent under conditions of negative buoyancy.
3. We validate that coordinated dual-fin propulsion can serve as a unified mechanism to flexibly generate diverse motion patterns, offering a novel approach for the design of minimalist underwater robots.

The remainder of the paper is organized as follows. Section 2 details the design of the bionic ocean sunfish robot, encompassing its mechanical structure and dual-fin cooperative strategy. Section 3 presents the experimental results of the robot’s two-dimensional motions and analyzes its swimming performance. Subsequently, Section 4 investigates its three-dimensional locomotion and discusses the robot’s excellent vertical maneuverability. In Section 5, a comprehensive discussion synthesizes the findings of these experiments. Finally, Section 6 provides concluding remarks.

## 2. Robot Design and Dual-Fin Actuation

**Figure 1:**
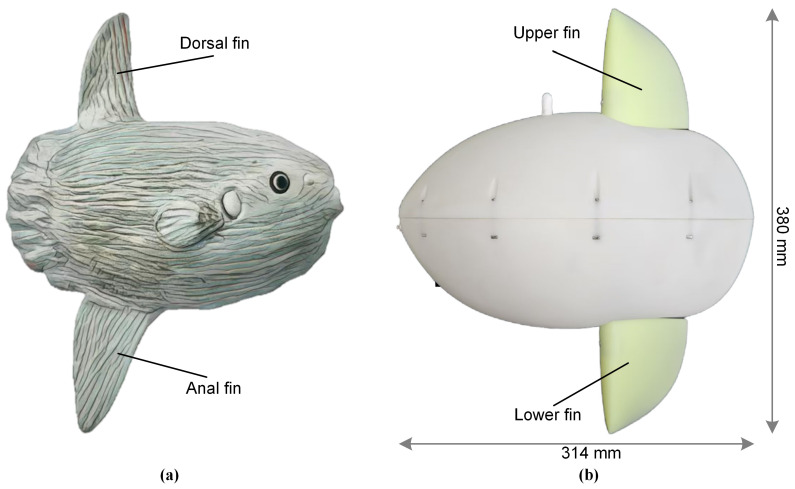
The ocean sunfish and bionic ocean sunfish robot. The shape and structure of (**a**) the ocean sunfish (hand-painted), and (**b**) the bionic ocean sunfish robot prototype.

The body of the ocean sunfish (*Mola mola*) is characterized by its large, truncated shape, lacking a conventional caudal fin. Its tail fin has atrophied, while its dorsal and anal fins are exceptionally long, capable of exceeding the body length when fully extended, as illustrated in the hand-drawn Figure 1a. The high stability and multiple motion modes of the ocean sunfish are attributed to these unique biological characteristics [27]. The dorsal and anal fins, positioned symmetrically on the vertical axis of the body, are crucial for generating both thrust and maneuverability [28]. The prototype robot, as shown in Figure 1b, is modeled after the ocean sunfish, adhering to principles of bionic design and modularity. The robot has a mass of 3322 g and overall dimensions of 314 mm in length, 124 mm in maximum width, and 380 mm in height (including fins).

### 2.1. Robot Prototype Design

As shown in Figure 1b, the robotic fish is composed of two main parts: an upper and a lower part, which are similar in appearance. The body shape features elliptical profiles in both height and thickness to minimize hydrodynamic drag. The robot’s shell and fins are fabricated from resin material using stereolithography (SLA) 3D printing (Prismlab Rapid 400), a method that offers a waterproof, low-cost, and rapid manufacturing process. Despite its relatively simple structure, the robot can achieve multiple motion modes through the coordinated action of its two vertically aligned fins.

**Figure 2:**
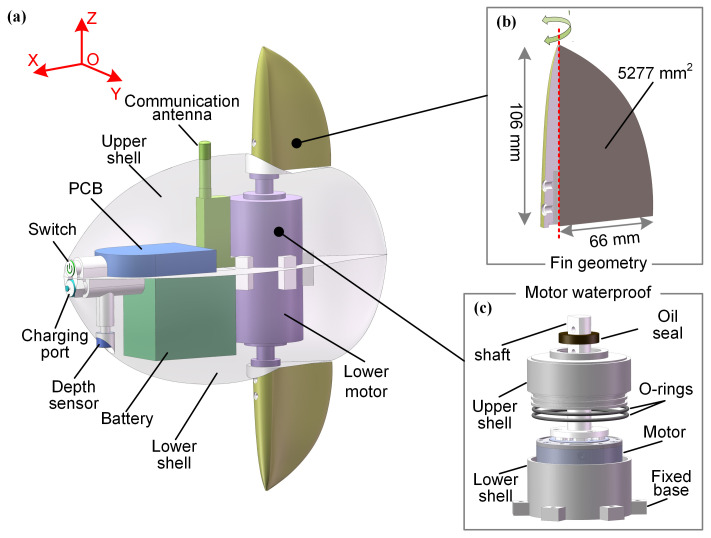
The internal structure details of the robot. (**a**) The three-dimensional structure of the bionic ocean sunfish robot. (**b**) The flap fin geometry design. (**c**) The motor waterproof design.

The internal structure of the robot is detailed in Figure 2. Two drive motors are positioned at the center of the robotic fish, providing thrust via fins connected by aluminum alloy shafts. These motors are independently controlled and secured to the upper and lower shells. To improve passive stability, the lightweight printed circuit board (PCB) and wireless communication module are located in the upper part, while the heavier battery module and depth sensor are placed in the lower part. This arrangement lowers the robot’s center of gravity, thereby minimizing body oscillations during movement. All internal modules are housed in their own waterproof enclosures. A waterproof power switch is located at the front of the robot, alongside a charging/programming interface connected via aviation plugs.

The fins are driven by two brushless direct current (DC) motors (LK-TECH MG4010E-i10) with built-in planetary reducers. These motors are selected for their high torque (peak torque of 4.5 N·m) and high speed (maximum speed of 320 rpm), which provide the necessary performance for dynamic motion. Communication between the control board and motors is established using the RS485 protocol, allowing independent control of each motor. The two motors are positioned in the upper and lower parts of the robot, respectively. The motors are housed in specially designed waterproof compartments, sealed with dual O-rings and sealant at critical points such as screw holes and wiring outlets. The transmission shaft is dynamically sealed with an oil seal, enhancing modularity for easy replacement and reducing the risk of water leakage. The waterproof motor assembly is shown in Figure 2c, and its effectiveness was validated in subsequent experiments. The fins are rigidly connected to the motors without any intermediate gear structure, a design choice intended to reduce system failures caused by mechanical wear.

**Figure 3:**
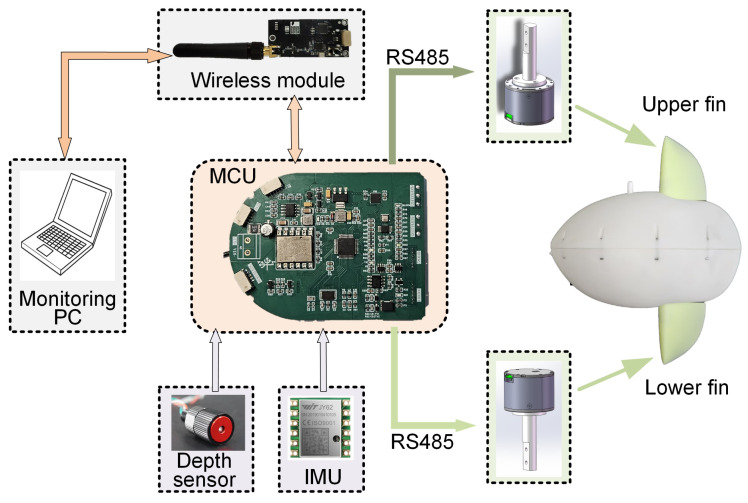
The electronics architecture of the robotic fish.

The robot’s motion control and task scheduling are handled by a custom control circuit board equipped with an STM32F103 microcontroller. This board processes data from two primary sensors: (i) a 6-axis inertial measurement unit (IMU, Xsens JY61P) located inside the body, providing data on acceleration, angular velocity, and three-dimensional orientation; and (ii) a depth sensor (TE MS5837-30BA), which is a pressure sensor installed at the front of the robot to measure depth. Additionally, the robot includes a flash memory module for logging sensor data and motor feedback. After processing by the STM32 chip, these data can be transmitted to a monitoring computer via the wireless module for subsequent analysis. All electronics and both motors are powered by a 6-cell (24 V, 4000 mAh) Li-ion battery with a DC-DC voltage regulator. Charging is performed through the aviation plug. To ensure continuous operation in water, the firmware can be updated and feedback date can be retrieved wirelessly, in addition to wired connections. The electronics architecture of the robotic fish is illustrated in Figure 3.

Detailed parameters of the robotic fish and control unit are provided in Table 1.

### 2.2. Dual-Fin Cooperation Strategies

A spatial Cartesian coordinate system is defined with the positive X-axis as the forward direction, the Y-axis as the lateral direction, and the Z-axis as the vertical direction. The height of a single fin is 106 mm (54.6% of the body height), which is similar to the fin-to-body height ratio of a biological ocean sunfish. The effective sweeping area of the fins is 5277 mm^2^ (10.8% of the body’s cross-sectional area in the X-Z plane), as shown in Figure 2b.

Each of the dorsal (upper) fin and anal (lower) fin is actuated by an independent motor, enabling the generation of thrust in different directions through coordinated flapping modes. The two motors rotate back and forth during the motion cycle, driving the fins to flap. The flapping motion of the fins is defined by sinusoidal oscillations of the motors’ rotation angles, as described below:

(1) $$\left\{\begin{array}{c} \theta_{p}\left(t\right)=A_{p}sin\left(2\pi f_{p}t+\varphi_{p}\right)+k_{p},\\ \theta_{b}\left(t\right)=A_{b}sin\left(2\pi f_{b}t+\varphi_{b}\right)+k_{b}, \end{array}\right.$$

where *t* is the time, θ is the angle of motor rotation (subscript *p* and *b* denote the upper and lower fins, respectively). The flapping range of the fins is limited to ±60° relative to the central plane. The motion is defined by four parameters: *A* is the flapping amplitude, *f* is the flapping frequency, φ is the initial phase, and *k* is an angular offset. The offset *k* is primarily applied to generate turning motions and is set to zero during cruising-straight.

Each motor is governed by these four parameters (A,f,φ,k), creating a total of eight control parameters for the dual-fin system. The robot’s locomotion is governed by distinct dual-fin coordination strategies for two-dimensional (2-D) and three-dimensional (3-D) motion.

For 2-D motion, the control parameters for the upper and lower fins are typically symmetric, with identical flapping frequencies and amplitudes. Specifically, we implement two distinct gait for forward swimming: in-phase flapping and anti-phase flapping, the latter of which is defined by a phase difference of $\varphi_{p}-\varphi_{b}=0∥\pi$. Turning maneuvers are executed by introducing an angular offset to the baseline flapping motions of the fins.

In contrast, 3-D motion is achieved through fully asymmetric control of the fins, enabling the robot to perform complex maneuvers that typically requires a greater number of actuators. The robot accomplishes diving by actuating only the lower fin and proactive ascent by actuating only the upper fin. Furthermore, a 3-D spiral motion is generated by combining the flapping of one fin with a static angular offset.

Typical parameter setting for these dual-fin coordination strategies are detailed in Table 2, with the underlying fluid dynamics discussed in Appendix A.

## 3. 2-D Motion Experimental Results

### 3.1. Experiment Setup

**Figure 4:**
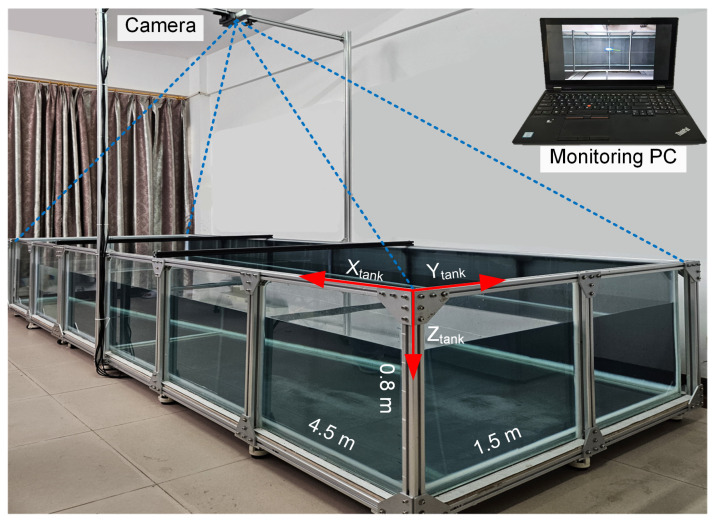
The water tank and overhead camera setup for tracking the robot’s 2-D trajectory and measuring its velocity.

Experiments evaluating the cruising-straight and turning maneuvers of the robotic fish were conducted in a custom-built water tank (4.5 m × 0.75 m × 0.8 m) filled with fresh water to a depth of 0.5 m. The tank is framed with aluminum profiles and mounted on caster wheels for mobility. To facilitate observation, the tank features transparent tempered glass sides. An overhead camera, mounted on an aluminum frame 2.0 m above the tank’s center, was used to capture the robot’s trajectory in the X-Y plane, as shown in Figure 4. The robot’s average forward speed was calculated from the recorded video by dividing the total distance traveled by the elapsed time. Additionally, both the movement trajectory and the video footage are saved during the measurement process. The 2-D tracking system was validated using stationary targets at known locations, yielding an average displacement error of less than 0.05 m. This error is considered negligible relative to the robot’s body length (BL), particularly in long-distance trials.

In addition to trajectory tracking, the onboard IMU was used to assess the robot’s dynamic stability. Specifically, the yaw amplitude (the maximum angle of the robot’s oscillation around the Z-axis) and roll amplitude (the maximum angle of the robot’s oscillation around the X-axis) were measured to quantify stability during different flapping gaits. To ensure data reliability for subsequent experiments, the IMU is calibrated before each trial. The IMU is reset upon power-on and then calibrated statically, resulting in an angular error of less than 0.2°. All experiments were conducted with the robotic fish in a free swimming, untethered configuration.

### 3.2. Cruising-Straight Experiments

Leveraging its vertically symmetrical dual-fin design, the robotic fish can propel itself forward using two distinct gaits: (i) in-phase flapping, where both fins move in the same direction ($\varphi_{p}-\varphi_{b}=0$), and (ii) anti-phase flapping, where the fins move in opposite directions ($\varphi_{p}-\varphi_{b}=\pi$). For all cruising-straight experiments, the flapping frequency and amplitude were kept identical for both fins ($A_{p}=A_{b},\;f_{p}=f_{b}$), and the angular offset was set to zero ($k_{p}=k_{b}=0$). A series of experiments were performed to evaluate the swimming speed and stability across 5 different flapping frequencies and 5 amplitudes, resulting in 25 total parameter combinations, with each combination being tested three times.

**Figure 5:**
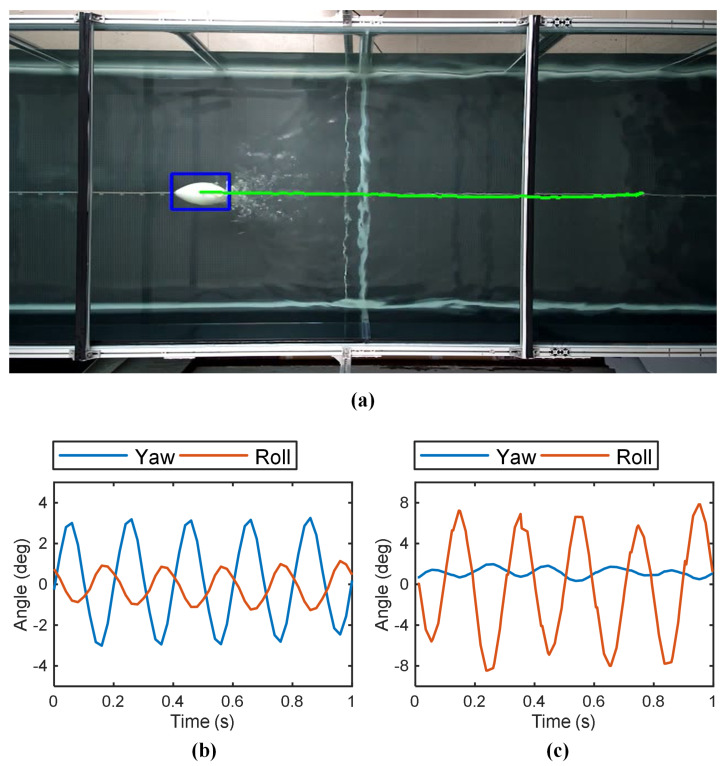
The cruising-straight motion of the robotic fish (A=50, f=5 Hz). (**a**) The cruising-straight trajectory (green line) of the robot fish (blue box) in the tank. Yaw and roll angle curves with (**b**) in-phase flapping gait and (**c**) anti-phase flapping gait.

The robot’s swimming speed was calculated from the overhead 2-D tracking system. Figure 5a shows a video snapshot of the robotic fish cruising-straight in the water tank at a certain moment. Its motion stability was evaluated using orientation data logged by the onboard IMU. During motion, the yaw and roll angles showed periodic oscillations over time, shown in Figure 5b,c. A smaller oscillation amplitude corresponds to higher motion stability. The yaw and roll amplitudes are defined as the average of the peak absolute values with their respective oscillation curves.

(1) In-phase flapping gait

**Figure 6:**
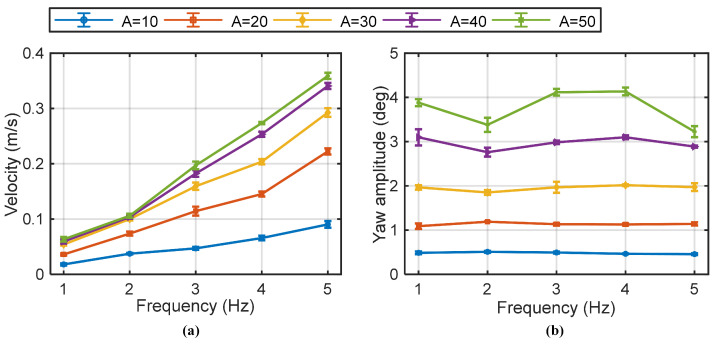
Robotic fish swimming velocity and yaw amplitude with in-phase flapping gait. (**a**) Swimming velocity, and (**b**) yaw amplitude during motions. Data points represent the mean of three independent experiments (N = 3 trials), with error bars indicating the standard deviation. Some error bars are smaller than the data markers, signifying minimal variance across measurements.

Figure 6a presents the swimming speed of the robotic fish at various flapping frequencies and amplitudes during in-phase flapping. As indicated in the figure, the swimming speed increases monotonically with both flapping frequency and amplitude, a trend consistent with biological observations of fish swimming in nature [26,29]. A maximum swimming speed of 0.365 m/s (1.16 BL/s) was recorded at a frequency of 5 Hz and an amplitude of 50°. This maximum tested speed does not necessarily represent the robot’s peak capability, as the parameter range was limited by the specifications of the drive motors and the mechanical design. It is hypothesized that higher frequencies could yield greater swimming speed, but at the expense of increased power consumption and reduced endurance. Figure 6b illustrates the robot’s stability during in-phase flapping, where yaw was the dominant form of instability, while roll was negligible. The robot maintained high stability at lower flapping amplitudes, but stability decreased as the amplitude increased. Interestingly, yaw amplitude was less pronounced at higher flapping frequencies, indicating that frequency and amplitude have opposing effects on yaw stability.

(2) Anti-phase flapping gait

**Figure 7:**
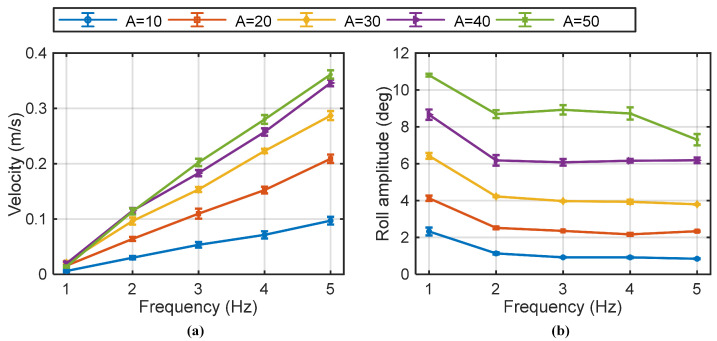
Robotic fish swimming velocity and roll amplitude with anti-phase flapping gait. (**a**) Swimming velocity, and (**b**) roll amplitude during motions (N = 3 trials).

Figure 7a illustrates the swimming speed and roll instability associated with anti-phase flapping gait. The relationship between swimming speed and the control parameters (flapping frequency, amplitude) followed a similar trend to the in-phase gait. At low flapping frequencies and amplitudes, the speed achieved with anti-phase flapping was notably lower than with in-phase flapping. However, as flapping frequency and amplitude increased, the performance gap between the two gaits diminished. A key characteristic of this gait is that the yaw was effectively suppressed (to less than 1°, as shown in Figure 5c) due to the symmetrical counter-motion of the fins, resulting in superior directional stability in the X-Y plane. However, this gait introduced significant body roll. Increasing the flapping amplitude was found to increase the magnitude of the roll, while roll was largely insensitive to changes in flapping frequency, as shown in Figure 7b. In summary, the anti-phase gait offered superior yaw stability at the cost of inducing roll. At higher performance parameters, both gaits could achieve comparable forward speeds, presenting a clear trade-off between yaw and roll stability.

### 3.3. Cruising-Turn Experiment

Turning maneuvers are achieved by activating the offset angle to the base in-phase flapping motion of both fins. This common offset directs the resultant thrust vector at an angle to the robot’s centerline, generating the yaw moment required for the turn. A smaller turning radius can enhance the maneuverability of the robotic fish [7]. In this study, we systematically investigated how varying this common offset angle *k* and the flapping amplitude *A* affects the robot’s turning performance. The key performance metrics—turning radius and turning angular velocity—present a classic trade-off that is crucial for practical applications.

**Figure 8:**
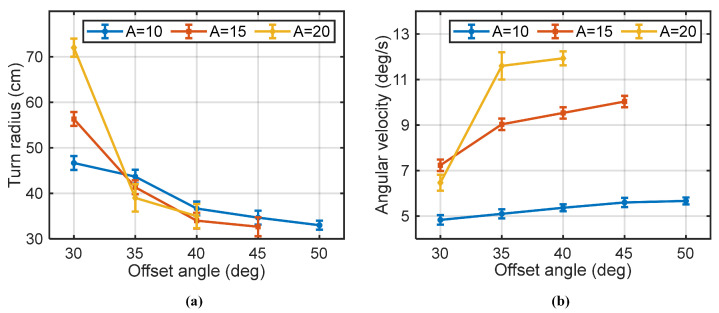
Turning radius and turning angular velocity at different offset angles (f=3 Hz, A+k≤60). (**a**) The turning radius. (**b**) The turning angular velocity (N = 3 trials).

The experimental results, shown in Figure 8, illustrate these relationships. Increasing the offset angle consistently leads to a smaller turning radius and a higher angular velocity. For instance, at large offset angles, the turning radii for all tested amplitudes converge to a minimum of approximately 0.30 m (0.96 BL). The effect of flapping amplitude is more nuanced. While a larger amplitude always results in a higher turning angular velocity, its impact on the turning radius depends on the offset. At small offsets, a high amplitude can undesirably increase the turning radius because the strong forward thrust component overpowers the weak turning moment. Therefore, achieving a tight turn requires a careful balance of a large offset angle with a moderate amplitude.

**Figure 9:**
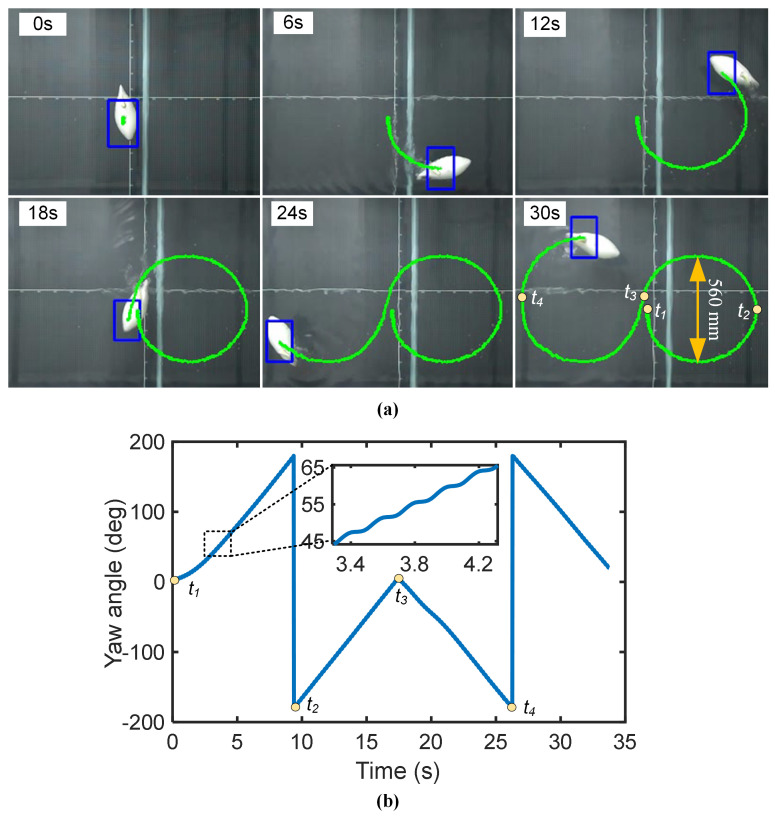
The turning motion of the robotic fish: it turns left from the starting point and swims a circle, then turns right and swims another circle. (**a**) The snapshot of the turning trajectory (green line). (**b**) The yaw angle curve of the turning (180° = −180°).

To demonstrate continuous steering control, the robot was programmed to execute a figure-eight trajectory, as shown in Figure 9a. The maneuver was initiated by setting a common offset of *k* = −40° for a left turn, followed by switching to *k* = +40° for a right turn. With a flapping frequency of 5 Hz and an amplitude of 15°, the robot achieved a stable turning radius of 0.28 m (0.89 BL) and a turning angular velocity of 20.6°/s. The corresponding linear velocity was 0.114 m/s (0.36 BL/s). This reduction from straight-line cruising speed is expected, as a portion of the propulsive thrust is redirected to generate the yaw moment. The yaw angle curve for this maneuver is presented in Figure 9b. These results confirm that the control parameters can be effectively tuned to achieve a desired compromise between turning radius and speed, highlighting the robot’s high maneuverability.

## 4. 3-D Motion Experimental Results

While conventional robotic fish often rely on auxiliary mechanisms for three-dimensional control, such as pectoral fins [13,30], center-of-gravity shifting [17], or buoyancy adjustment systems [16]. These methods invariably increase mechanical complexity. In contrast, the proposed robotic fish accomplishes complex 3-D maneuvers using only its two primary fins, eliminating the need for additional structures.

### 4.1. Experiment Setup

**Figure 10:**
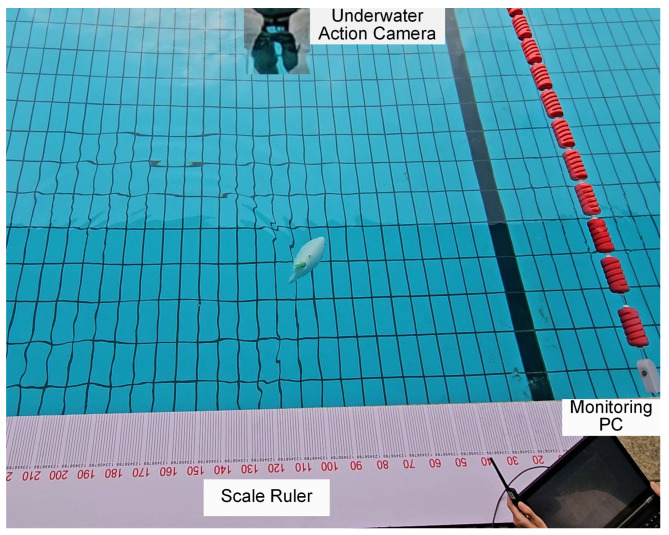
The 3-D motion experimental environment: an outdoor swimming pool.

The three-dimensional motion experiments were conducted in an outdoor swimming pool with a depth of 131 cm, as shown in Figure 10. The robot’s movements were captured by an underwater action camera (GoPro HERO 12) and a second camera positioned above the water surface. Snapshots illustrating the descending, ascending, and spiral motions were extracted from the recorded video footage.

Real-time depth data were logged by the onboard depth sensor. The sensor, calibrated in the atmosphere before each trial to ensure an measurement accuracy of better than 0.2 cm. The sensor recorded a total vertical travel distance of 96 cm between the water surface (reading 20 cm) and the pool bottom (reading 116 cm). Due to the significant attenuation of wireless signals underwater, communication was lost when the robot’s antenna submerged beyond approximately 40 cm. Consequently, all 3-D motion routines were pre-programmed, and the sensor data were stored on the onboard flash memory for post-mission retrieval and analysis.

### 4.2. Descending and Proactive Ascending

The robot achieves vertical motion by actuating a single fin, which generates both a vertical force and forward thrust. This allows for controlled descents and proactive ascents without any dedicated control surfaces.

(1) Activate the lower fin to descend

For a positively buoyant robot to descend, the lower fin is actuated to generate a downward thrust sufficient to overcome the net buoyant force. A series of experiments at a fixed frequency (5 Hz) revealed a clear relationship between flapping amplitude and diving capability. An amplitude below 20° was insufficient to initiate a descent. However, at *A* = 25°, an average descent speed of 6.4 cm/s was achieved, and this speed increased proportionally with amplitude, reaching 17.8 cm/s at *A* = 50°.

**Figure 11:**
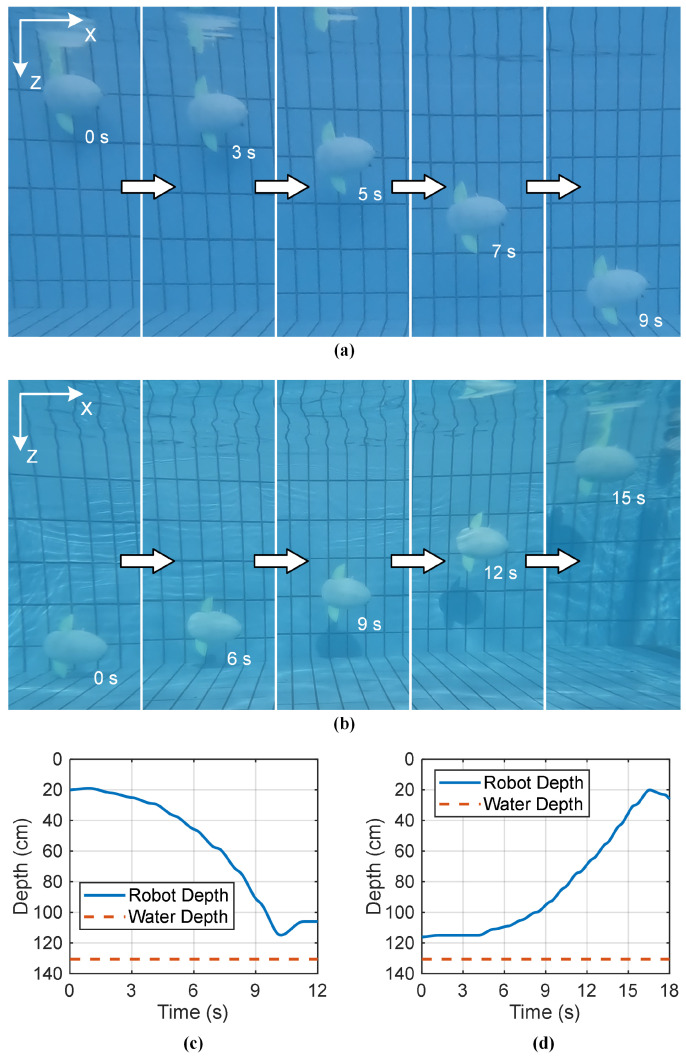
Snapshot and depth information of the robotic fish performing a descent and an ascent. Snapshot of the robot performing (**a**) descent, and (**b**) ascent. Depth curve of the robot performing (**c**) descent, and (**d**) ascent.

A representative descent is shown in Figure 11a. With Only the lower fin flapping at *f* = 5 Hz and *A* = 30° (the upper fin remains inactive), the robotic fish successfully dove from the water surface to the pool bottom (a 96 cm descent and a 250 cm forward distance) in 9.6 s. The corresponding depth curve, shown in Figure 11c, exhibits a distinct acceleration phase, confirming that the downward thrust generated by the lower fin exceeded the robot’s positive buoyancy.

(2) Activate the upper fin to proactively ascend

A key capability of the robot is its ability to proactively ascend even when negatively buoyant, a crucial feature for bottom-based operations and recovery. To validate this, a small weight was added to make the robot sink naturally.

From the bottom, activating only the upper fin (*f* = 5 Hz and *A* = 30°) generated the upward thrust needed for ascent, as illustrated in Figure 11b.The robot ascended from the pool bottom to the water surface in 16.1 s, covering a forward distance of 437 cm. The depth curve in Figure 11d shows a acceleration, confirming that the upward thrust generated by the upper fin successfully overcame the robot’s negative buoyancy.

**Figure A1:**
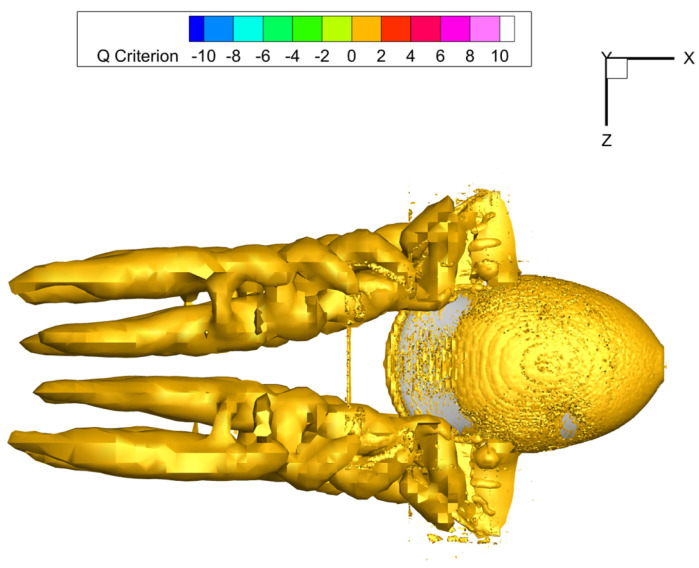
Three dimensional vortex structure of the dual-fin robotic fish in a CFD environment.

In both maneuvers, the single-fin actuation generates simultaneous vertical and forward thrust, a mechanism supported by the vortex structures shown in Figure A1. Throughout these motions, the robot also maintained a stable posture (see the Video in Supplementary File), a testament to the inherent stability of its design.

### 4.3. Spiral Motion

The preceding experiments demonstrate that the robot can execute discrete motions such as cruising, turning, descending, and ascending. By strategically combining these capabilities, the robot can achieve complex three-dimensional trajectories. This section details the generation and control of a spiral descent, which is achieved by superimposing a turning moment onto a single-fin-driven descent.

To systematically analyze this maneuver, we investigated how different fin offset strategies affect the spiral radius and descent rate, with results summarized in Table 3. The baseline motion was a descent driven solely by the lower fin, flapping at 5 Hz with a 30° amplitude. Three distinct turning strategies were then applied: (i) upper fin as a static rudder: applying a 30° fixed offset to the inactive upper fin; (ii) lower fin with dynamic offset: applying a 30° offset to the flapping motion of the active lower fin; (iii) combined offset: applying a 30° offset to both the active lower fin and the inactive upper fin.

The results in Table 3 show that a combined offset produces the tightest turn, with a spiral radius of 54 cm and a descent rate of 9.6 cm/s. In contrast, using only the inactive upper fin as a rudder resulted in the widest spiral (radius 116 cm). This demonstrates that in addition to frequency and amplitude, the method of applying fin offsets provides a versatile tool for fine-tuning the spiral trajectory.

**Figure 12:**
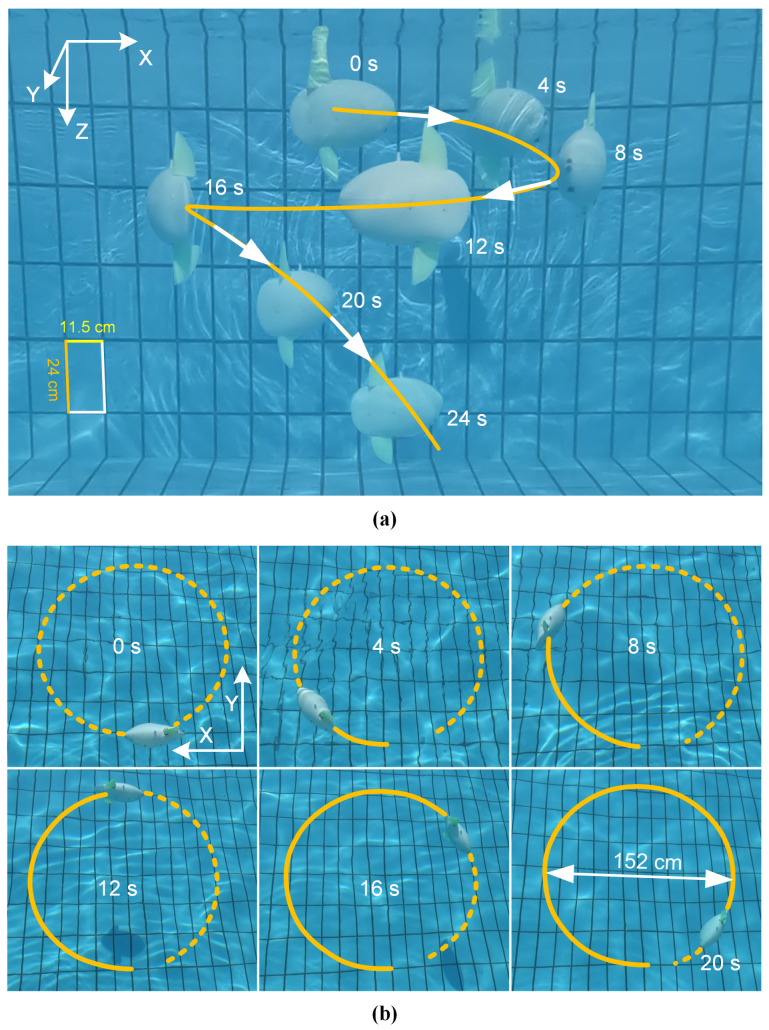
Snapshot of the robotic fish performing a three-dimensional spiral descent. (**a**) Side view and (**b**) top view of the spiral trajectory (orange line) of the robotic fish.

A complete spiral descent is illustrated in Figure 12. For this demonstration, the robot was positively buoyant. The lower fin was activated with a frequency of 4 Hz and an amplitude of 25°, while the inactive upper fin was held at a fixed 30° angle to act as a rudder, and the active lower fin also flapped with a 30° offset. As shown in Figure 12a,b, the robot executed a continuous, stable spiral to the pool bottom in 24 s, achieving a measured radius of 76 cm. This trajectory can be further optimized; the spiral radius can be tightened by adjusting the offset angles, and the descent speed can be increased by increasing the flapping frequency or amplitude of the propulsive fin.

## 5. Discussion

In this work, we have designed and validated a bionic dual-fin robotic fish that achieves a comprehensive suite of maneuvers using only two actuators. The foundation of its versatility lies in its core design: the vertical, symmetric arrangement of the two fins. This unique mechanical structure, when combined with specific, coordinated control strategies, provides direct control over forces in both the horizontal and vertical planes. It is this deliberate design choice that unlocks the robot’s rich, multi-modal locomotion without resorting to any auxiliary control mechanisms. This approach allows for a direct mapping between fin kinematics and the resulting motion, forming the basis for all demonstrated capabilities.

The performance of the robot, enabled by this design, is highly competitive. Its maximum cruising-straight speed of 1.16 BL/s compares favorably to many flapping-based platforms, such as those by Berlinger et al. [31] (0.6 BL/s), Chen et al. [5] (1.07 BL/s), Clark et al. [32] (0.63 BL/s), and Bonnet et al. [33] (1 BL/s). While slightly slower than the 1.21 BL/s [12] achieved by the dual-caudal-fin robotic fish (which was achieved by a large wingspan), our design offers superior postural stability, with a yaw amplitude of less than 4.2°, a critical advantage for sensor-based tasks. Regarding turning, while its agility does not match that of multi-joint robots which utilize body flexion for rapid C-shaped turns [9,16,17], it demonstrates a key advantage over its rigid-bodied counterparts. It achieves a small turning radius (0.89 BL) without the pectoral fins that are often required in other designs to improve maneuverability [12,31]. The turning radius of the flapping-based robotic fish can be reduced by pectoral fins, which will also increase the complexity of the system. Our robotic fish can achieve a small turning radius without pectoral fins, and adding more actuators will be a matter of our future work. In the vertical plane, the same design principle allows for controlled descents and, most notably, proactive ascents from a negatively buoyant state through the simple asymmetric actuation of a single fin.

The most significant implication of this work is the validation of a minimalist design philosophy: achieving functional complexity through control rather than mechanical accumulation. These 3-D experiments demonstrate that our dual-fin robot exhibits robust maneuverability in the vertical plane, a feat that requires more actuators in other robots—typically four actuators in [13,16,17,31], or three in [34,35,36]. This radical simplification of the mechanical system not only enhances reliability and reduces potential points of failure but also maximizes internal volume for energy storage and scientific payloads, offering a distinct advantage for long-duration, real-world missions.

To provide a clearer and more direct comparison, the key performance metrics of our robot are benchmarked against several representative robotic fish from the literature in Table 4. This comparison focuses on critical aspects such as cruising speed, turning agility, and the underlying mechanical complexity for achieving three-dimensional motion. This contextualization highlights the unique advantages of our dual-fin cooperative mechanism.

Despite the successful validation of its core capabilities, we acknowledge several limitations that define the trajectory for future work. The experiments, particularly for 3-D motions, were conducted in an open-loop fashion using pre-programmed routines. Furthermore, our analysis focused on kinematic performance, lacking a systematic study of the energy efficiency across different gaits and maneuvers. Lastly, all tests were conducted in a controlled, static pool environment. Future research will therefore prioritize the development of closed-loop autonomous control by leveraging onboard sensor feedback, conducting a thorough optimization of energy efficiency, and validating the robot’s robustness and utility through deployment in challenging, real-world aquatic environments.

## 6. Conclusions

In this study, we presented the design and validation of a bionic ocean sunfish robot, that utilizes a unique vertical dual-fin propulsion system. The central achievement of this work is the demonstration of a comprehensive suite of 2-D and 3-D maneuvers—including high-speed cruising (up to 1.16 BL/s), agile turning (minimum radius of 0.89 BL), and controlled vertical motion—–using only two actuators and no auxiliary control mechanisms. We have established that the robot maintains exceptional postural stability (yaw amplitude of less than 4.2°) and, most notably, can achieve both descents and proactive ascents from a negatively buoyant state through the asymmetric actuation of a single fin. This validates our core design philosophy: achieving complex, multi-modal locomotion through specific, coordinated control strategies rather than increased mechanical complexity.

The results confirm that this mechanically simple yet functionally sophisticated design offers a robust and reliable alternative to conventional underwater robots that depend on multiple actuators and auxiliary control mechanisms. While the current implementation relies on open-loop control for complex maneuvers, the demonstrated capabilities establish a strong foundation for future research. Our immediate future work will focus on developing closed-loop autonomous control by leveraging onboard sensor feedback, enabling the robot to perform complex tasks such as path following and target tracking in dynamic environments. Ultimately, the proposed dual-fin robot represents a promising platform with significant potential for long-duration applications in marine science and exploration.

## Figures and Tables

**Table 1:** Parameters of the robot and control unit.

Items	Characteristics
Dimensions	314 mm (L) × 124 mm (W) × 380 mm (H)
Mass	3322 g
Motors	2 × 4.5 N·m
Micro-controller unit	STM32F103
Communication module	433 MHz
Battery	22.2 V-6S-4000 mAh
Attitude sensor	JY61P
Depth sensor	MS5837-30BA

**Table 2:** Parameter settings for different swimming motions.

Swimming Motions	Flapping Amplitude	Flapping Frequency	Initial Phase	Angular Offset
Cruising	$A_{p}=A_{b}\neq 0$	$f_{p}=f_{b}\neq 0$	$\varphi_{p}-\varphi_{b}=0∥\pi$	$k_{p}=k_{b}=0$
Turning	$A_{p}=A_{b}\neq 0$	$f_{p}=f_{b}\neq 0$	$\varphi_{p}=\varphi_{b}=0$	$k_{p}∥k_{b}\neq 0$
Descending	$A_{b}>0=A_{p}$	$f_{b}>0=f_{p}$	$\varphi_{p}=\varphi_{b}=0$	$k_{p}=k_{b}=0$
Proactive ascending	$A_{p}>0=A_{b}$	$f_{p}>0=f_{b}$	$\varphi_{p}=\varphi_{b}=0$	$k_{p}=k_{b}=0$
Spiraling descend	$A_{b}>0=A_{p}$	$f_{b}>0=f_{p}$	$\varphi_{p}=\varphi_{b}=0$	$k_{p}∥k_{b}\neq 0$
Spiraling ascend	$A_{p}>0=A_{b}$	$f_{p}>0=f_{b}$	$\varphi_{p}=\varphi_{b}=0$	$k_{p}∥k_{b}\neq 0$

**Table 3:** Comparison of spiral radius and diving velocity of three distinct offset strategies (*f* = 5 Hz, *A* = 30° and *k* = 30°, N = 3 trials).

Offset Fin(s)	Average Descending Speed	Average Spiral Radius
Upper fin	7.7 cm/s (0.25 BL/s)	116 cm (3.69 BL)
Lower fin	9.1 cm/s (0.29 BL/s)	94 cm (2.99 BL)
Double fins	9.6 cm/s (0.31 BL/s)	54 cm (1.72 BL)

**Table 4:** Comparison with other representative robotic fish.

Robotic Fish	Max. Speed (Locomotion Mode)	Min. Turning Radius	3-D Motion/ Actuators
Boxfish-like robot [5]	1.07 BL/s (MPF)	Not specified	Yes/4
Dual caudal fins robot [12,13]	1.21 BL/s (BCF)	0.23 BL (with pectoral fins)	Yes/4
SoFi [16]	0.5 BL/s (BCF)	1.66 BL	Yes/4
Bionic sailfish robot [17]	1.6 BL/s (BCF)	0.39 BL (with dorsal fin)	Yes/4
Sailfish robot [34]	0.54 BL/s (BCF)	2.16 BL	Yes/3
Snapp [35]	1.7 BL/s (BCF)	0.96 BL	Yes/3
Finbot [31,37]	1 BL/s (BCF)	1.2 BL/0.5 BL (without/with pectoral fins)	Yes/4
Robotic shark [38]	0.8 BL/s (BCF)	0.2 BL (with pectoral fins)	Yes/7
Our robotic fish	1.16 BL/s (MPF)	0.89 BL (without auxiliary fins)	Yes/2

## Data Availability

The original contributions presented in this study are included in the article. Further inquiries can be directed to the corresponding author.

## Supplementary Materials

The following supporting information can be downloaded at: https://www.mdpi.com/article/10.3390/biomimetics10080489/s1, Video S1: Experimental Video of the Bionic Ocean Sunfish Robot.

## Appendix A. Wake Vortex Structure in CFD

To elucidate the hydrodynamic principles underpinning the robot’s propulsion, this appendix presents a computational fluid dynamics (CFD) analysis of the wake structure. Figure A1 illustrates the three-dimensional vortex structures, identified by the Q-criterion (iso-surface at Q = 1), generated during the acceleration phase with in-phase flapping at *f* = 3 Hz and *A* = 30°.

A critical observation is that, unlike conventional caudal fins that produce vortex rings aligned primarily with the direction of motion, our vertical dual-fin system generates a vectored thrust with a significant vertical component. This unique orientation of the shed vortices provides the hydrodynamic basis for the robot’s multi-modal swimming and is fundamental to the three-dimensional maneuvering capabilities demonstrated in this paper.
